# Tocilizumab treatment for COVID-19 patients: a systematic review and meta-analysis

**DOI:** 10.1186/s40249-021-00857-w

**Published:** 2021-05-18

**Authors:** Qiu Wei, Hua Lin, Rong-Guo Wei, Nian Chen, Fan He, Dong-Hua Zou, Jin-Ru Wei

**Affiliations:** 1 Department of Respiratory and Critical Care, The Fifth Affiliated Hospital of Guangxi Medical University, Nanning, Guangxi 530022 People’s Republic of China; 2 Clinical Medical Laboratory Center, The Fifth Affiliated Hospital of Guangxi Medical University, 89 Qixing Road, Nanning, 530022 Guangxi China; 3 Department of Infectious Diseases, The Fifth Affiliated Hospital of Guangxi Medical University, Nanning, Guangxi 530022 People’s Republic of China; 4 Department of Hematology, School of Medicine, Washington University in St Louis, St. Louis, MO 63130 USA

**Keywords:** Tocilizumab, COVID-19, IL-6, Cytokine storm

## Abstract

**Background:**

Coronavirus disease 2019 (COVID-19) has killed over 2.5 million people worldwide, but effective care and therapy have yet to be discovered. We conducted this analysis to better understand tocilizumab treatment for COVID-19 patients.

**Main text:**

We searched major databases for manuscripts reporting the effects of tocilizumab on COVID-19 patients. A total of 25 publications were analyzed with Revman 5.3 and R for the meta-analysis. Significant better clinical outcomes were found in the tocilizumab treatment group when compared to the standard care group [odds ratio (*OR*) = 0.70, 95% confidential interval (*C*): 0.54–0.90, *P* = 0.007]. Tocilizumab treatment showed a stronger correlation with good prognosis among COVID-19 patients that needed mechanical ventilation (*OR* = 0.59, 95% *CI*, 0.37–0.93, *P* = 0.02). Among stratified analyses, reduction of overall mortality correlates with tocilizumab treatment in patients less than 65 years old (*OR* = 0.68, 95% *CI*: 0.60–0.77, *P* < 0.00001), and with intensive care unit patients (*OR* = 0.62, 95% *CI*: 0.55–0.70, *P* < 0.00001). Pooled estimates of hazard ratio showed that tocilizumab treatment predicts better overall survival in COVID-19 patients (*HR* = 0.45, 95% *CI*: 0.24–0.84, *P* = 0.01), especially in severe cases (*HR* = 0.58, 95% *CI* 0.49–0.68, *P* < 0.00001).

**Conclusions:**

Our study shows that tocilizumab treatment is associated with a lower risk of mortality and mechanical ventilation requirement among COVID-19 patients. Tocilizumab may have substantial effectiveness in reducing mortality among COVID-19 patients, especially among critical cases. This systematic review provides an up-to-date evidence of potential therapeutic role of tocilizumab in COVID-19 management.

**Graphical abstract:**

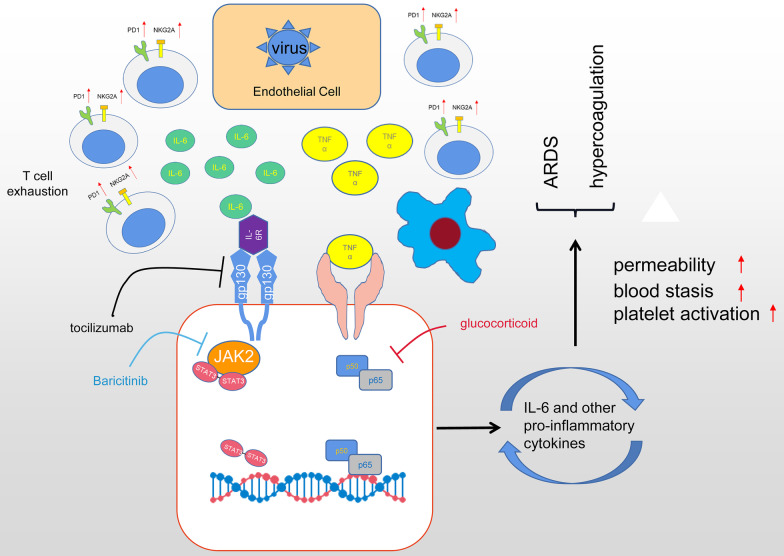

**Supplementary Information:**

The online version contains supplementary material available at 10.1186/s40249-021-00857-w.

## Background

The outbreak of coronavirus disease 2019 (COVID-19) has spread rapidly worldwide and became a global pandemic. [1]. As of April 29, 2021, more than 149 million confirmed cases of COVID-19 have been reported to the World Health Organization (WHO), including more than 3.1 million deaths. [2].

COVID-19 can cause symptoms ranging from mild to very severe, most of COVID-19 patients present mild infection and can recover within weeks. Those who show clinical features of pneumonia, respiratory failure, acute respiratory distress syndrome (ARDS), hypercoagulability or septic shock, require hospitalization for management [3]. Although the pathogenesis of COVID-19 remains unclear, an accumulating body of evidence suggests that hyperinflammation with overproduction of pro-inflammatory cytokines is frequently observed in severe COVID-19 patients and presumably contribute to a poor prognosis [4, 5]. Elevated serum cytokines, including interleukin-6 (IL-6), IL-10, tumor necrosis factor-α (TNF-α) and interferon-γ, may cause fatal ARDS and coagulation disorders in COVID-19 patients [6]. In particular, serum interleukin-6 elevation is strongly associated with COVID-19 severity and mortality [7]. Thus, the inhibition of IL-6 is hypothesized to be a promising therapeutic strategy to interfere with COVID-19-induced cytokine storm and thereby alter the course of disease progression.

Tocilizumab, a recombinant humanized anti-IL-6 receptor monoclonal antibody, has been approved for uses in patients with rheumatologic disorders and chimeric antigen receptor T cell-induced cytokine release syndrome. Recent publications revealed clinical benefits of tocilizumab treatment in COVID-19 patients [8, 9]. The role of IL-6 inhibition in reducing COVID-19 severity and mortality, however, remains controversial because several large-scale, multi-center observations and randomized clinical trials show minimal benefits [10, 11]. It is necessary to systematically evaluate and update the effects of IL-6 inhibition among COVID-19 patients as new data are generated. Previous meta-analysis investigating tocilizumab were published before a few large, randomized control trials [12, 13]. We included all eligible publications up to this point, investigating the impact of tocilizumab on reducing mortality and intubation in COVID-19 patients.

## Methods and materials

### Literature search

This meta-analysis was conducted according to the Preferred Reporting Items for Systematic Reviews and Metanalysis (PRISMA) guidelines (Additional file 1: Table S1). Two authors (QW, HL) independently searched English publications in PubMed, PMC, Scopus, Google Scholar, and Web of Science. The first active search was performed on December 27, 2020, while the last was performed on March 20, 2020. We used the following keywords and the combinations in the query: “novel coronavirus” or “COVID-19” or “SARS-CoV-2” and “tocilizumab” or “IL-6 blockade” or “IL-6 receptor antagonist”. We retrieved all the references in all manuscripts for future analysis.

### Selection criteria

Manuscripts were selected if they were (1) English, peer-reviewed journal articles, (2) studies reporting tocilizumab treatment in COVID-19 patients, (3) studies assigning COVID-19 patients to severity classes, (4) only studying adult patients, (5) patients’ mortality data was available in the paper. Only the most recent study was included if the same investigator published multiple studies using the same dataset.

### Quality assessment

Three authors (QW, HL and RGW) assessed the entry manuscripts according to the principles adapted from Xu et al. [14]. The following items were evaluated in the assessment: the clarity of study objectives; whether or not there was a clearly stated study period (start date and end date); the description clarity of the patient selection criteria; whether the study was international or national; the stated tocilizumab treatment method and dose; whether the baseline equivalence groups were clearly considered; the definition of the primary outcome (overall mortality or mechanical ventilation requirement) prior to the study; if the follow-up period was long enough (# months); whether a clear hazard ratio (*HR*) with 95% confidence intervals (95% *CI*) was stated; and the limitations of each study were considered. We ranked the selected papers according to the quality items used in each study (score range 0–10). Quality assessment was not used as exclusion criteria.

### Data extraction

We extracted the following information from each included study in this meta-analysis: first author, study period, country, study countries, study type (retrospective or prospective), total number of patients, sex ratio in each group, age in each group, tocilizumab treatment, clinical outcomes (overall mortality and mechanical ventilation requirement), tocilizumab group positive and negative outcome numbers, control group positive and negative outcomes, and *HR* corresponding 95% *CI* if available.

### Statistical analysis

Revman 5.3 (The Nordic Cochrane Centre, Copenhagen, Denmark) and R programming language (http://www.R-project.org/) were used to analyze the data. During the full-paper screening process, Cohen’s kappa statistic was used to evaluate the inter-reviewer agreement. Subgroup analyses were also used to study how study areas, patient’s median age, patient’s severity, study size, and male percentage in the groups would affect tocilizumab treatment outcomes. The publication biases were assessed through Begg’s funnel plot in Revman 5.3 and Egger's test in R. Sensitivity analysis was conducted in R to find potential outliers by omitting one study at a time (also called the “one-study removed” model). Statistical heterogeneity between studies was determined by Cochran’s Q test and Higgins *I* square where *P* < 0.1 or *I*^*2*^ > 50% was considered as high heterogeneity and a random-effect model was used to combine the data; otherwise, a fixed-effect model was used. A two-sided *P* value less than 0.05 was considered statistically significant.

## Results

### Literature search

**Fig. 1:**
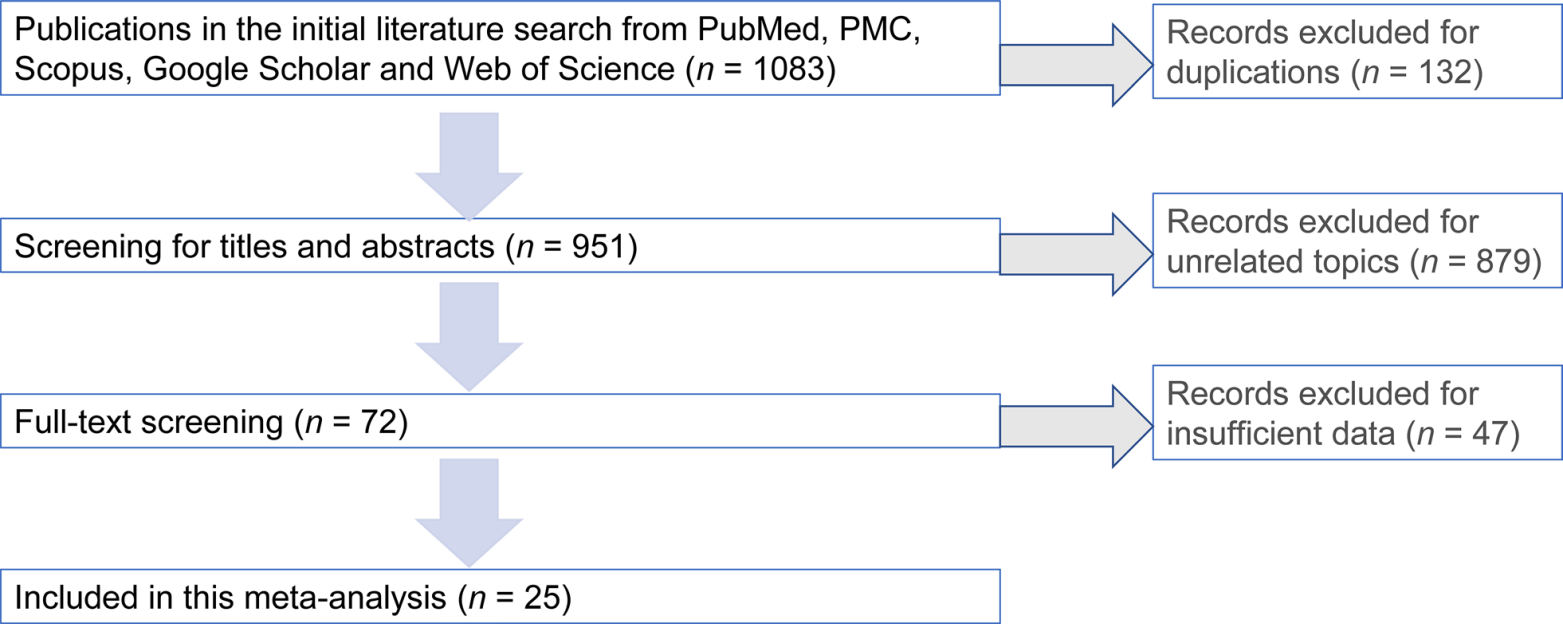
Flow-diagram of this meta-analysis

Our literature search flow chart is shown as Fig. 1. We had a total of 1083 publications in the initial literature search. After removing duplicate records, 951 were screened for more details. After scanning the titles and abstracts, 72 were included in the full-text screening. After reviewing the included papers carefully, 47 publications were excluded for insufficient data, leaving 25 publications for this meta-analysis. Cohen's kappa for inter-reviewer agreement was 0.78 for title and abstract screening and 0.81 for full-text screening. The quality of the 25 included publications was fair with an average quality score of 7.72 and a median score of 8 (range 5–10, Additional file 2: Table S2).

### Literature details

Overall, 10 201 patients from 25 different publications were included in this study. Seven of the studies assessed patients from the USA [10, 15–20], 13 of the studies assessed patients from west Europe [8, 21–32], two assessed patients from multiple countries [33, 34], one assessed patients from Brazil [35], and two studies were from India [36, 37]. The main characteristics of the 25 included studies were summarized in Table 1.

**Table 1:** COVID-19 patients characteristics with IL-6 inhibitor Tocilizumab treatment effects

Record	First author	Study period	Country	Study type	Total cases	Sex(M) T group	Sex(M) SOC	Age (T group)	Age (SOC)	Outcomes	T group positive outcome	SOC positive outcome	T group negative outcome	SOC negative outcome	HR (95% *CI*)	Tocilizumab treatment
1	Martínez-sanz	31/01/20–23 /04/20	Spain	Retrospective	1229	191 (73%)	574 (59%)	65 (55–76)*	68 (57–80)*	Overall mortality	61	120	199	849	0.34 (0.17–0.71)	A median total dose of 600 mg
1	Martínez-sanz	31/01/20–23 /04/20	Spain	Retrospective	286	NA	NA	NA	NA	Overall mortality (crp > 150 mg/dl)	NA	NA	NA	NA	0.39 (0.19–0.80)	
2	Potere	28/03/20–21/04/20	Italy	Retrospective case-control	80	26 (65.0%)	26 (65.0%)	56.0 (50.3–73.2)	54.5 (50.0–73.0)	Overall mortality	2	11	38	29	NA	324 mg, two concomitant subcutaneous injections
3	Canziani	23/02/20–09/05/20	Italy	Retrospective case-control	128	47 (73%)	47 (73%)	63 (12)*	64 (8)*	30-day mortality	17	24	47	40	0.61 (0.33–1.15)	Two intravenous infusion of 8 mg/kg
3	Canziani	23/02/20–09/05/20	Italy	Retrospective case-control	128	48 (73%)	48 (73%)	64 (12)*	65 (8)	Mechanical ventilation requirement at 30 days	9	29	55	35	0.36 (0.16–0.83)	
4	Tsai	01/03/20–05/05/20	USA	Retrospectiv	132	46(69.7%)	50(75.8%)	62.38 ± 13.49*	61.35 ± 16·09*	Overall mortality	18	18	48	48	NA	800 mg or 600 mg or 400 mg; one or two doses
5	Roomi	01/03/20–30/05/20	USA	Retrospective	176	60 (72.30)	23 (27.70)	65.48*	58.09	Overall mortality	6	13	26	131	NA	
6	Guaraldi	21/02/20–30/04/20	Italy	Retrospective	544	127 (71%)	232 (64%)	64 (54–72)	69 (57–78)	Overall mortality	13	73	166	292	0·38 (0·17–0·83)	Two intravenous infusion of 8 mg/kg (up to 800 mg), 12 h apart
6	Guaraldi	21/02/20–30/04/20	Italy	Retrospective	544	127 (71%)	232 (64%)	64 (54–72)	69 (57–78)	Mechanical ventilation requirement	33	57	146	308	0·61 (0·40–0·92)	
7	Menzella	11/03/20–14/04/20	Italy	Retrospective	79	29 (71%)	27 (71%)	63.3 ± 10.6*	70.3 ± 11.3*	Overall mortality	10	20	31	18	0.55 (0.22–1.35)	Two intravenous infusion of 8 mg/kg (up to 800 mg), 12 h apart, or subcutaneously 2 to 4 doses of 162 mg
7	Menzella	11/03/20–14/04/20	Italy	Retrospective	79	29 (71%)	27 (71%)	63.3 ± 10.6*	70.3 ± 11.3*	Mechanical ventilation requirement	9	12	32	26	0.44 (0.22–0.89)	
8	Salvarani	31/03/20–11/06/20	Italy	Prospective randomized clinical trial	123	40 (66.7%)	37 (56.1%)	61.5 (51.5–73.5)	60.0 (54.0–69.0)	Overall mortality at 30 days	2	1	58	62	NA	Two intravenous infusion of 8 mg/kg (up to 800 mg), 12 h apart.
9	Gupta	04/03/20–10/05/20	USA	Retrospective	3924	299 (69.1%)	2165 (62.0%)	58 (48–65)	63 (52–72)	Overall mortality	125	1419	308	2072	0.71 (0.56–0.92)	Intravenously or subcutaneously during the first 2 days of icu admission
10	Marte	08/03/20–25/04/20	USA	Retrospective	193	74 (77.1%)	63 (64.9%)	58.8 ± 13.6*	62.0 ± 14*	Overall mortality	43	55	53	42	NA	One dose only
11	Klopfenstein	01/03/20–13/04/20	France	Retrospective	45	NA	NA	76.8 (52–93) ± 11*	70.7 (33–96) ± 15*	Overall mortality	5	12	15	13	NA	One or two doses
11	Klopfenstein	01/03/20–13/04/20	France	Retrospective	45	NA	NA	76.8 (52–93) ± 11*	70.7 (33–96) ± 15*	Mechanical ventilation	0	8	20	17	NA	
12	Biran	01/03/20–22/04/20	USA	Retrospective	630	155 (74%)	281 (67%)	62 (53–71)	65 (56–74)	Hospital-related mortality	102	256	108	164	0.71 (0.56–0.89)	Intravenous infusion of 400 mg or 8 mg/kg
13	Campochiaro	Na	Italy	Retrospective	65	29 (91%)	27 (82%)	64 (53 – 75)	60 (55 – 75.5)	Overall mortality at 28th day	5	11	27	22	NA	Two intravenous infusion of 400 mg
14	Capra	26/02/20–02/04/2020	Italy	Retrospective	85	45(73%)	19 (83%)	63 (54–73)	70 (55–80)	Overall mortality	2	11	60	12	0.035 (0.004–0.347)	400 mg iv once, or subcutaneous 324 mg once or 800 mg iv once
15	Stone	20/04/20–15/06/20	USA	Prospective randomized clinical trial	243	96 (60%)	45 (55%)	61.6 (46.4–69.7)	56.5 (44.7–67.8)	Mechanical ventilation or death at 28 day	17	10	144	72	0.83 (0.38–1.81)	A single dose of 8 mg/kg intravenously, up to 800 mg
15	Stone	20/04/20–15/06/20	USA	Prospective randomized clinical trial	243	96 (60%)	45 (55%)	61.6 (46.4–69.7)	56.5 (44.7–67.8)	Mechanical ventilation at 28 day	11	8	150	74	0.65 (0.26–1.62)	
16	Kaminski	26/03/20–18/05/20	USA	Retrospective	125	38(58%)	45(75%)	58.9 ± 17.9*	57.2 ± 15	Overall mortality at 28 day	24	30	41	30	NA	400 mg as a single dose iv or an subcutaneous dose of 324 mg
17	Eimer	11/03/20–15/04/20	Sweden	Retrospective	87	28 (96.6%)	45 (77.6%)	58.0 (49.0–63.0)	55.0 (52.0–64.8)	30‐day all‐cause mortality	5	19	24	39	0.52 (0.19–1.39)	A single dose of 8 mg/kg intravenously
17	Eimer	11/03/20–15/04/20	Sweden	Retrospective	87	28 (96.6%)	45 (77.6%)	58.0 (49.0–63.0)	55.0 (52.0–64.8)	Mechanical ventilation	24	53	5	5	NA	
18	Salama	Until 30/09/2020	International	Prospective randomized clinical trial	377	150 (60.2%)	73 (57.0%)	56.0 ± 14.3*	55.6 ± 14.9*	Mechanical ventilation or death by day 28	30	25	219	103	0.56 (0.33–0.97)	Two doses of intravenous (8 mg/kg, up to 800 mg)
18	Salama	Until 30/09/2020	International	Prospective randomized clinical trial	377	150 (60.2%)	73 (57.0%)	56.0 ± 14.3*	55.6 ± 14.9*	Death at day 28	26	11	223	117	NA	
19	Remap–cap	Until 17/06/2020	UK	Prospective randomized clinical trial	755	261 (74%)	283 (70%)	61.5 ± 12.5*	61.1 ± 12.8*	In–hospital death	98	142	252	255	0.56(0.33–0.97)	8 mg/kg (up to 800 mg)
20	Rosas	03/04/20–28/05/20	International	Prospective	438	205 (69.7%)	101 (70.1%)	60.9 ± 14.6*	60.6 ± 13.7*	Death at day 28	58	28	236	116	0.3 (-7.6 to 8.2)	8 mg/kg (up to 800 mg)
21	Hermine	31/03/20–18/04/20	France	Prospective	130	44 (70%)	44 (66%)	64.0 (57.1–74.3)	63.3 (57.1–72.3)	Death at day 28	7	8	56	59	0.92 (0.33–2.53)	8 mg/kg (up to 800 mg) at day1; an additional fixed dose of tcz, 400 mg on day 3
22	Veiga	08/05/20–17/06/20	Brazil	Prospective	129	44 (68)	44 (69)	57.4 ± 15.7*	57.5 ± 13.5*	Death at day 28	14	6	51	58	NA	A single intravenous infusion at 8 mg/kg
22	Veiga	08/05/20–17/06/20	Brazil	Prospective	129	44 (68)	44 (69)	57.4 ± 15.7*	57.5 ± 13.5*	Mechanical ventilation requirement at day 29	4	4	61	60	NA	A single intravenous infusion at 8 mg/kg
23	Soin	30/05/20–31/08/20	India	Prospective	180	76 (84%)	76 (86%)	56 (47–63)	54 (43–63)	Death at day 28	11	15	80	73	NA	A single dose between 4 mg/kg and 8 mg/kg plus an additional dose if required
23	Soin	30/05/20–31/08/20	India	Prospective	180	76 (84%)	76 (86%)	56 (47–63)	54 (43–63)	Incidence of mechanical ventilation at day 28	14	13	77	75	NA	A single dose between 4 mg/kg and 8 mg/kg plus an additional dose if required
24	Albertini	06/04/20–21/04/20	France	Retrospective	44	16 (73%)	15 (68%)	64 (41–80)	65 (41–82)	Death at day 14	1	0	21	22	NA	A fixed dose of 600 mg for patients <100 kg and 800 mg for those >100 kg
24	Albertini	06/04/20–21/04/20	France	Retrospective	44	16 (73%)	15(68%)	64 (41–80)	65 (41–82)	Mechanical ventilation requirement at day 14	2	6	20	16	NA	A fixed dose of 600 mg for patients <100 kg and 800 mg for those >100 kg
25	Gokhale	31/03/20–05/07/20	India	Retrospective	269	107 (70.9%)	69 (58.5%)	53 (44–60)	55 (47–64)	Overall mortality	79	74	72	44	NA	A single intravenous infusion of 400mg

*HR* hazard ratio, *NA* Not applicable,* SOC* Standard of care, *Age was described in mean ± standard deviation (when other studies used median and interquartile range)

### Meta-analysis results

#### Tocilizumab’s overall effect on clinical outcomes

**Fig. 2:**
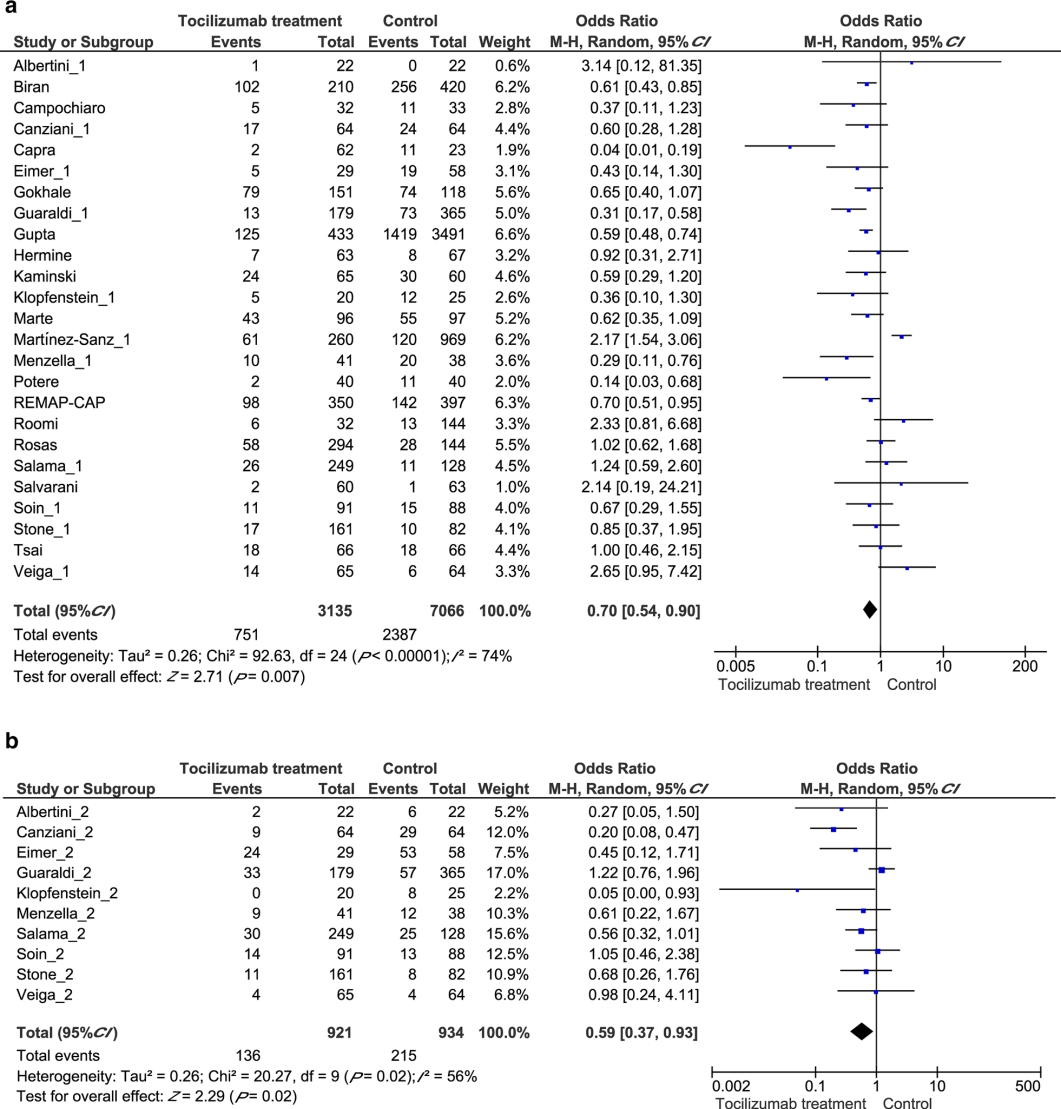
Forest plots displaying pooled odds ratios (*OR*s) for Tocilizumab treatment on different outcomes. **a** the pooled *OR* for Tocilizumab treatment on all clinical outcomes; **b** the pooled *OR* for Tocilizumab treatment on mechanical ventilation requirement outcome. *CI*: Confidence interval

First, we performed analyses to evaluate tocilizumab’s effect on overall mortality and mechanical ventilation requirement. Our pooled analysis revealed a significant difference between the tocilizumab group (715/3135, 22.8%) and control group (2387/7066, 33.8%) in the random-effect model [Odds ratio (*OR*) = 0.70, 95% confidential interval (*CI)*: 0.54–0.90, *P* = 0.007, Fig. 2a], suggesting efficacy of tocilizumab treatment for COVID-19. We also analyzed the studies focused on mechanical ventilation and tocilizumab treatment significantly reduced the requirement for mechanical ventilation (*OR* = 0.59, 95% *CI*: 0.37–0.93, *P* = 0.02, Fig. 2b).

#### Subgroup analysis

We conducted various stratified analyses to identify possible confounders in tocilizumab treatment studies. First, we divided the manuscripts into different categories according to their traits including (1) study location: USA vs west Europe; (2) age differences: reported mean/median age older than 65 vs younger than 65; (3) disease severity: ICU patients vs general patients; (4) study sizes: patient group size of 150 or less vs 151 and more; (5) gender imbalance: studies with 10% more males in the tocilizumab treatment group than the control group vs 10% or less males.

**Fig. 3:**
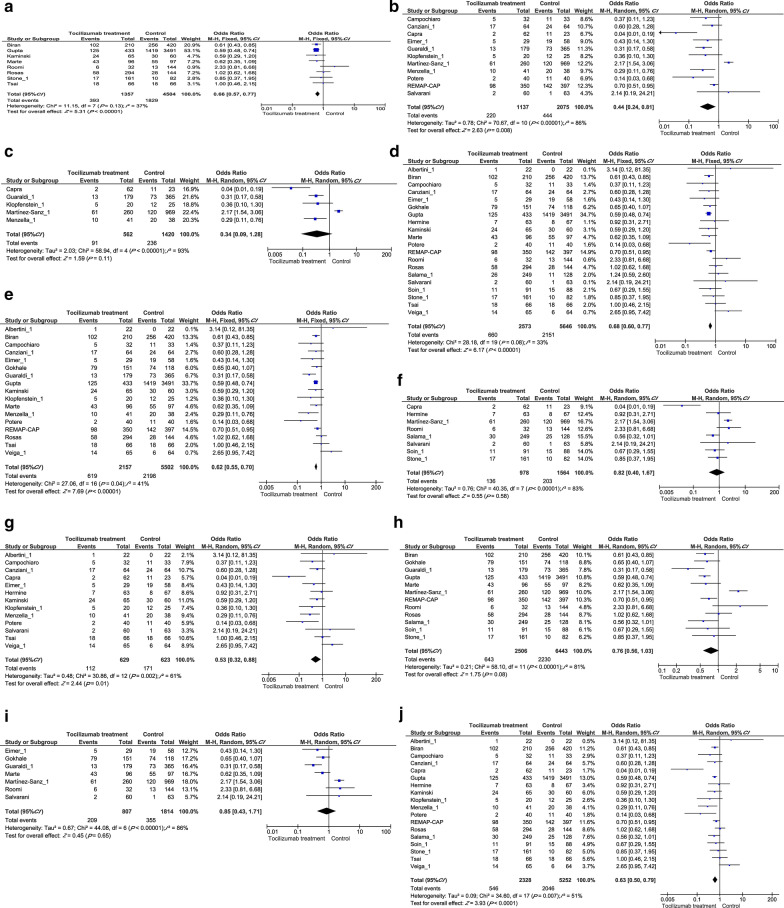
Subgroup analysis of pooled *OR* for Tocilizumab treatment on COVID-19 prognosis. **a** USA patients; **b** West Europe patients; **c** patients with a median age ≥ 65; **d** patients with a median age < 65; **e** ICU patients **f** general patients **g** total patient number less than 150 **h** total patient number more than 150; **i** studies that had 10% more male in the treatment group than the control group **j** studies that has balance male percentage in treatment and control group. *CI* Confidence interval

In both USA and western Europe, tocilizumab treatment significantly reduced mortality (*OR* = 0.66, 95% *CI*: 0.57–0.77, *P* < 0.00001, Fig. 3a; and *OR* = 0.44, 95% *CI*: 0.24–0.81, *P* = 0.008, Fig. 3b), other regions are too little studies to make a subgroup. Tocilizumab treatment did not show efficacy among older subpopulations (*OR* = 0.34, 95% *CI*: 0.09–1.28, *P* = 0.11, Fig. 3c), but significantly benefited patients whose mean/median age is less than 65 years old (*OR* = 0.68, 95% *CI*: 0.60–0.71, *P* < 0.00001, Fig. 3d). Because we divided studies based on their reported median/mean age, results are based on characteristics of the whole group and not the individuals within. Our results also showed that tocilizumab treatment significantly improved outcome among severe or ICU-admitted COVID-19 patients (*OR* = 0.62, 95% *CI*: 0.55–0.70, *P* < 0.00001, Fig. 3e), but had no effects on general COVID-19 patients (*OR* = 0.82, 95% *CI*: 0.40–1.67, *P* = 0.58, Fig. 3f). Interestingly, tocilizumab treatment significantly improved outcomes in studies with 150 patients or less (*OR* = 0.53, 95% *CI*: 0.32–0.88, *P* = 0.01, Fig. 3g), but not in larger studies (*OR* = 0.76, 95% *CI*: 0.56–1.03, *P* = 0.08, Fig. 3h). Tocilizumab treatment did not correlate with improved clinical outcome in male dominated studies (*OR* = 0.85, 95% *CI*: 0.43–1.71, *P* = 0.65, Fig. 3i), but associates with improved outcomes in gender-balanced studies (*OR* = 0.63, 95% *CI*: 0.50–0.79, *P* < 0.0001, Fig. 3j), which suggests different responses to SARS-COV-2 between male and female patients.

#### Hazzard ratio on clinical outcomes

**Fig. 4:**
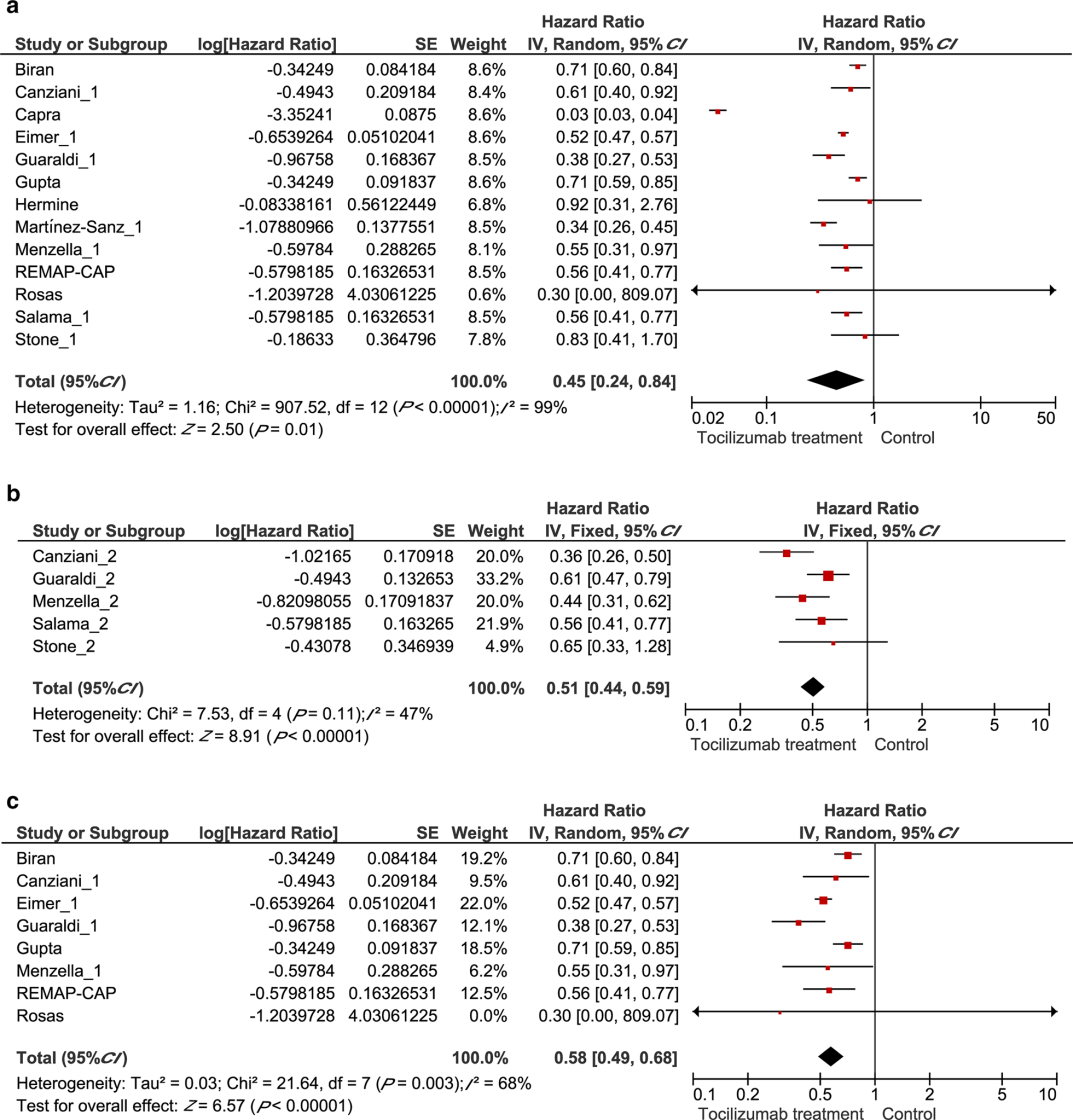
Forest plots displaying pooled hazard ratios (HRs) for Tocilizumab treatment on different outcomes. **a** the pooled HR for Tocilizumab treatment on all clinical outcomes; **b** the pooled HR for Tocilizumab treatment on mechanical ventilation requirement outcome; **c** the pooled HR for Tocilizumab treatment on overall mortality from ICU patients. *CI* Confidence interval

To further evaluate the prognostic effects of tocilizumab treatment among COVID-19 patients, we extracted the multivariate HRs and their 95% *CI* in these studies to calculate a combined HR, demonstrating that patients with a tocilizumab treatment had better outcomes than patients receiving standard care (*HR* = 0.45, 95% *CI*: 0.24–0.81, *P* = 0.01, Fig. 4a). In terms of the secondary outcome, tocilizumab was associated with a lower probability of requiring invasive ventilation (*HR* = 0.51, 95% *CI*: 0.44–0.59, *P* < 0.00001, Fig. 4b). Among severe COVID-19 patients, the administration of tocilizumab correlates with a markedly better prognosis (*HR* = 0.58, 95% *CI* 0.49–0.68, *P* < 0.00001, Fig. 4c).

### Meta-analysis quality control

**Fig. 5:**
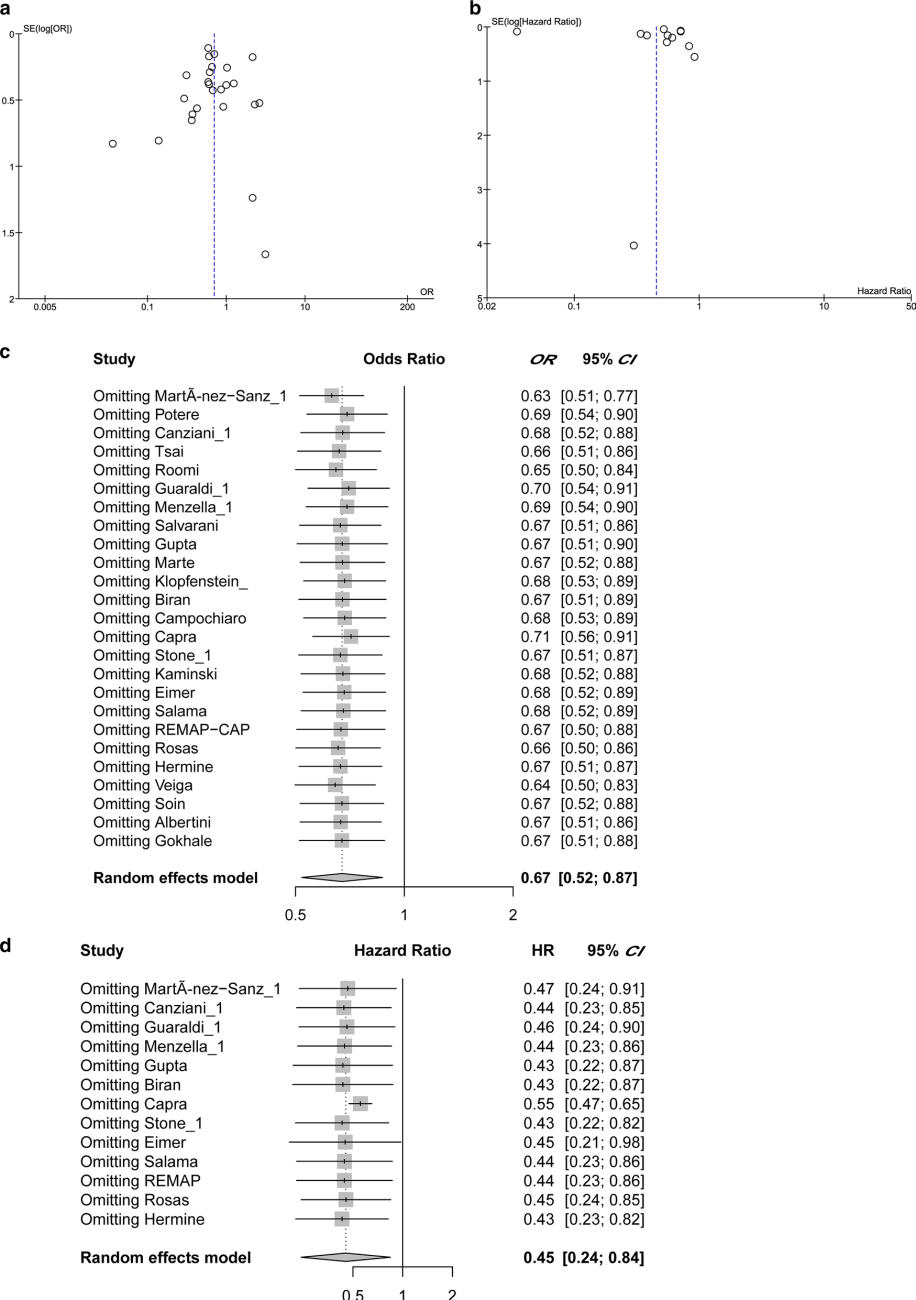
Quality assessment. **a** The funnel plots of all outcome odds ratio analysis. **b** The funnel plot of pooled hazard ratio analysis. **c** The sensitivity analysis of OR studies by omitting one at a time. **d** The sensitivity analysis of all HR studies. *CI* Confidence interval, *HR* Hazard ratio, *OR* Odds Ratio

Begg's funnel test was used to estimate all the existing publication bias of the literature in this meta-analysis. As shown in Fig. 5a, the shape of the funnel plots of all outcomes showed no evidence of asymmetry, with an Egger's test bias intercept at -0.41 (*P* = 0.58). For the hazard ratio analysis, Begg's funnel test does not show asymmetry (Fig. 5b), with an Egger’s test bias intercept of 0.4982 (*P* = 0.6184). The observed tocilizumab treatment effects on all outcomes (by *OR*) and prognosis (by *HR*) were not significantly affected by removing any one of the studies included, as is shown in Fig. 5c, d. In summary, there were no significant outliers in this meta-analysis.

## Discussion

In 2020, the spreading of COVID-19 brought unprecedented healthcare and economy costs and substantial morbidity globally. Major causes of deaths in severe COVID-19 patients were ARDS and disseminated intravascular coagulation, which result from uncontrolled inflammatory processes after SARS-CoV-2 infection [3]. Interestingly, SARS-CoV-2 invades airway epithelial cells without triggering the secretion of type I and III interferon, the first line of defense to block early virus replication [38]. Instead, the infected airway epithelia produce IL-6 and other pro-inflammatory cytokines, attracting monocytes and cytotoxic T cells to the infection site to recognize and to destroy the infected cells [39]. Then, macrophages are summoned to engulf the apoptotic cells through phagocytosis. In healthy responses, SARS-CoV-2 infections are resolved through this process, the level of inflammatory cytokines recedes, and patients recover [40]. In severely affected COVID-19 patients, however, excessive secretion of IL-6 and other pro-inflammatory cytokines summon T cell aggregation and cause T cell functional exhaustion with increased expression of PD-1 and NKG2A [41]. This is confirmed by the commonly observed correlation of lymphopenia with elevated cytokine profiles in severe COVID-19 patients [42]. Furthermore, hyper-secreted IL-6 will activate the JAK-STAT and NF-κB signaling pathways in both immune and non-immune cells, inducing a massive and sustained production of NF-κB target genes, including IL-6 and other chemokines [43]. Such a positive feedback loop of IL-6 secretion further fuels hyperinflammation and increases vascular permeability leading to pulmonary edema and ARDS. Moreover, the cytokine storm also results in the disruption of vascular endothelium, blood stasis and the activation of coagulation, triggering a hypercoagulable status in COVID-19 patients [44]. It has been well-recognized that COVID-19-induced cytokine storm is a critical contributor to COVID-19 relevant mortality [40, 45]. Controlling the COVID-19-induced cytokine storm is important for improving severe COVID-19 patients' prognosis.

Although there are no approved therapies for the COVID-19-induced cytokine storm, various strategies targeting different stages of the cytokine storm have been proposed. Glucocorticoid has powerful anti-inflammatory properties and was widely used during the outbreaks of SARS and MERS, but clinical evidence for corticosteroid treatment of SARS-CoV-2-induced lung injuries remains controversial. A large-scale clinical trial showed that the use of dexamethasone resulted in lower 28-day mortality among those severe patients, but the authors also warned that high doses or wrong administration timing can be harmful as glucocorticoid delays viral clearance [46]. Another study from Wuhan showed that high-dose corticosteroid uses were associated with death in patients with severe COVID-19 [3].

Given the pivotal role of IL-6 in COVID-19 induced cytokine storm, it is attractive to target hyperinflammation during SARS-CoV-2 infection via the blockage of IL-6. Tocilizumab is a competitive inhibitor of both the membrane-bound and soluble IL-6 receptor, preventing downstream signal transduction of IL-6. Early reports of tocilizumab treatment in COVID-19 patients showed promising results, while the lack of control and small sample sizes dampened their reliability[24, 25]. To address this question, several multi-center cohort studies inspected the efficiencies of tocilizumab on several subpopulations of COVID-19 patients. Their findings revealed a correlation of early Tocilizumab administration with lower mortality rates among critically ill COVID-19 patients with a rapid disease trajectory[19]. More importantly, Tocilizumab demonstrated satisfactory safety in clinics because COVID-19 patients receiving Tocilizumab do not show higher incidences of adverse events, including secondary infections and hepatotoxicity [33].

**Fig. 6:**
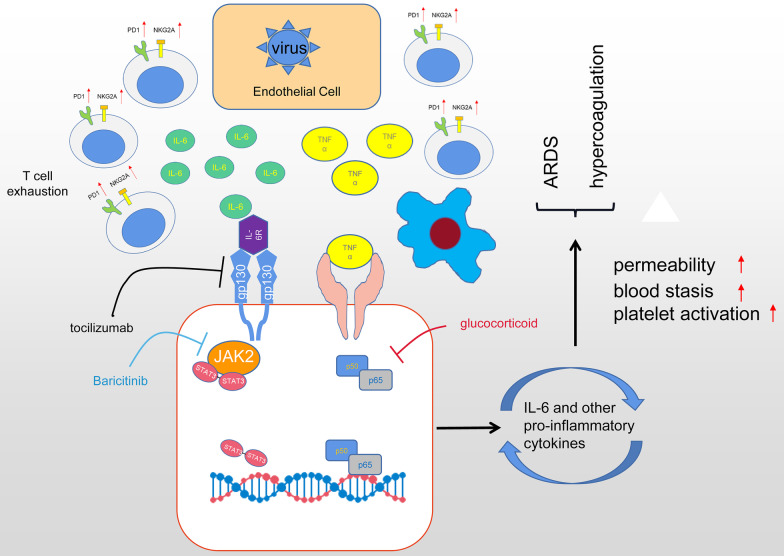
Mechanisms of SARS-CoV-2 associated cytokine storm and targeted therapeutic approaches

Another possible approach of COVID-19-induced cytokine storm mitigation is to inhibit the JAK-STAT intracellular signaling pathway. As we mentioned before, the activation of JAK-STAT and NF-κB signaling pathways are important mediators of cytokine storm by receiving signals from proinflammatory signals, such as IL-2, IL-6, IL-10, IFN-γ, and GM-CSF, and producing more proinflammatory cytokines. Baricitinib, an orally administered selective inhibitor of JAK 1 and 2, was found effective in reducing recovery time among COVID-19 patients, especially in severe cases requiring high-flow oxygen or noninvasive ventilation [47]. Baricitinib is also suggested to reduce viral entry due to its inhibition of AP2-associated protein kinase 1[48], however, the concerns of increasing viral loads and thromboembolism risks limit its uses. Taken together, the suppression of COVID-19 induced cytokine storms is key to the effective treatment of severe COVID-19 patients and can be targeted with different strategies. We summarized the possible mechanisms and therapeutic strategies to address the COVID-19-induced cytokine storm in Fig. 6.

In this study, we reported that the administration of tocilizumab to COVID-19 patients is associated with reduced mortality and shorter intubation time. However, the conclusion should be interpreted with caution since several randomized clinical trials fail to support it. There are some confounders that should contribute to the inconsistency. Most importantly, both the trials recruited general, but not severe, COVID-19 patients for study [19, 33]. According to our analysis, however, the association of tocilizumab with clinical benefits is even stronger among severe COVID-19 patients. The most recently published prospective randomized clinical trial backs our conclusion, suggesting that COVID-19 patients with moderate or severe disease were more likely to benefit from tocilizumab [33]. It is of great necessity to conduct more clinical trials to pinpoint the subgroups of COVID-19 patients that are most likely to benefit tocilizumab treatment. In addition, routes, dosing, and timing of tocilizumab administration also play important roles and need to be considered carefully. Currently, there are no standard regulations and doctors apply tocilizumab empirically or based on availability. Although the efficacy and safety of subcutaneous tocilizumab are comparable to intravenous tocilizumab in most clinical applications [49], intravenous tocilizumab is preferred over subcutaneous therapy to treat COVID-19-induced cytokine storm [20]. Currently, tocilizumab is administered either at fixed doses or dependent on bodyweight. Most of the patients received tocilizumab quickly after entering ICU, which presumably represents a hyperinflammatory prime. In addition, the lack of a reliable/useful/accurate biomarker for the COVID-19-induced cytokine storm dampens the efficiency of tocilizumab. Currently, serum IL-6 level, along with other inflammatory markers such as CRP, ferritin, are used in isolation or together to determine and predict the efficacy of tocilizumab in COVID-19 treatment. Further studies are warranted.

There are some advantages of this systematic review when compared to several published ones with the similar topic. Small-scale, unbalanced or non-peer-reviewed studies constitute the major sources of previous meta-analyses [12, 13]. The inclusion of recently published high impact large-scale studies enable us to provide more reliable and updated insights into tocilizumab uses in COVID-19 patients. Notably, to minimize the interferences of confounding factors, we conducted various subgroup analyses, such as severe patient only-, age stratified- and gender stratified- analyses. Our results demonstrated that severely ill COVID-19 patients can benefit more from tocilizumab treatment, providing evidence for further clinical trials and patient managements. We also evaluated the efficacy of tocilizumab uses on different outcomes, including mortality and the reduction in mechanical ventilation duration. There are some limitations in this study as dosing, timing and routes of tocilizumab administration vary among the included manuscripts. Furthermore, the definition of COVID-19 severity is inconsistent among the included manuscripts. Finally, the follow-up time in terms of mortality occurrence is not the same across all studies, which might result in immortal time bias to some degree.

## Conclusions

This meta-analysis included 25 peer-reviewed publications with more than 10 201 individuals to analyze the correlation of tocilizumab with clinical outcomes among COVID-19 patients. Our study shows that tocilizumab treatment is associated with a lower risk of mortality and mechanical ventilation requirement among COVID-19 patients, especially among the critically ill. A combined HR also demonstrated that COVID-19 patients receiving tocilizumab treatment had better prognosis than those receiving standard care. Our stratified sub-group analysis revealed that disease severity, age, and sex play important roles in determining the efficacy of tocilizumab. Therefore, our results provide substantial evidence that tocilizumab benefits critically ill COVID-19 patients.

## Supplementary Information

**Additional file 1: Table S1**. PRISMA 2009 Checklist.

**Additional file 2: Table S2**. Quality assessment of included studies.

## Data Availability

The dataset supporting the conclusion of this article is available from the corresponding author upon reasonable request.

## Abbreviations

ARDS: Acute respiratory distress syndrome
COVID-19: Coronavirus disease 2019
*CI*: Confidence interval
*HR*: Hazard ratio
ICU: Intensive care unit
IL-2: Interleukin-2
IL-6: InterleukinInterleukin-6
IL-10: InterleukinInterleukin-10
Jak1: Janus kinase 1
Jak2: Janus kinase 2
MERS: Middle east respiratory syndrome
NKG2A: Natural killer cell receptor group 2 member A
NF-κB: Nuclear factor-κB
*OR*: Odds Ratio
PD-1: Programmed-cell death protein 1
PRISMA: Preferred Reporting Items for Systematic Reviews and Meta Analyses
SARS: Severe acute respiratory syndrome
SARS-CoV-2: Severe acute respiratory syndrome coronavirus 2
STATs: Signal transducer and activator of transcription proteins
TNF-α: Tumor necrosis factor-α
