# Supplementary material for: Tocilizumab treatment for COVID-19 patients: a systematic review and meta-analysis
Source: Infect Dis Poverty. 2021 May 18;10:71. doi: 10.1186/s40249-021-00857-w (PMC8128625; doi:10.1186/s40249-021-00857-w)
Supplement: Supplementary file 2 — Additional file 2: Table S2. Quality assessment of included studies. [file 40249_2021_857_MOESM2_ESM.docx]

**Supplementary Table 2** Quality assessment of included studies.

| Record | First author | Study Objectives | Study Period | Patient Selection Criteria | International  Or multi centers | Tocilizumab Treatment | baseline equivalence groups | primary outcome | follow-up period | HR with 95% CI | limitations considered | Overall score |
| --- | --- | --- | --- | --- | --- | --- | --- | --- | --- | --- | --- | --- |
| 1 | Martínez-Sanz | Yes | Yes | Yes | No | Yes | Yes | Yes | No | Yes | Yes | 8 |
| 2 | Potere | Yes | Yes | No | No | Yes | Yes | Yes | Yes | No | No | 6 |
| 3 | Canziani | Yes | Yes | No | No | Yes | Yes | Yes | Yes | Yes | Yes | 8 |
| 4 | Tsai | Yes | Yes | No | No | Yes | Yes | Yes | Yes | Yes | Yes | 8 |
| 5 | Roomi | Yes | Yes | Yes | No | No | Yes | Yes | No | No | Yes | 6 |
| 6 | Guaraldi | Yes | Yes | Yes | No | Yes | Yes | Yes | No | Yes | Yes | 8 |
| 7 | Menzella | Yes | Yes | Yes | No | Yes | Yes | Yes | Yes | No | No | 7 |
| 8 | Salvarani | Yes | Yes | Yes | No | Yes | Yes | Yes | Yes | Yes | Yes | 9 |
| 9 | Gupta | Yes | Yes | Yes | No | Yes | Yes | Yes | Yes | Yes | Yes | 9 |
| 0 | Marte | Yes | Yes | Yes | No | Yes | Yes | Yes | No | No | Yes | 7 |
| 11 | Klopfenstein | Yes | Yes | No | No | Yes | Yes | Yes | No | No | No | 5 |
| 12 | Biran | Yes | Yes | Yes | No | Yes | Yes | Yes | Yes | Yes | Yes | 9 |
| 13 | Campochiaro | Yes | No | Yes | No | Yes | Yes | Yes | Yes | No | Yes | 7 |
| 14 | Capra | Yes | Yes | Yes | No | Yes | Yes | Yes | No | Yes | Yes | 8 |
| 15 | Stone | Yes | Yes | Yes | No | Yes | Yes | Yes | Yes | Yes | Yes | 9 |
| 16 | Kaminski | Yes | Yes | Yes | No | Yes | Yes | Yes | Yes | No | Yes | 8 |
| 17 | Eimer | Yes | Yes | No | No | Yes | Yes | Yes | Yes | No | Yes | 7 |
| 18 | Salama | Yes | Yes | Yes | Yes | Yes | Yes | Yes | Yes | Yes | No | 9 |
| 19 | REMAP-CAP | Yes | Yes | Yes | No | Yes | Yes | Yes | Yes | Yes | Yes | 9 |
| 20 | Rosas | Yes | Yes | Yes | Yes | Yes | Yes | Yes | Yes | Yes | Yes | 10 |
| 21 | Hermine | Yes | Yes | Yes | No | Yes | Yes | Yes | Yes | Yes | Yes | 9 |
| 22 | Veiga | Yes | Yes | Yes | No | Yes | Yes | Yes | Yes | No | Yes | 8 |
| 23 | Soin | Yes | Yes | Yes | No | Yes | Yes | Yes | Yes | No | Yes | 8 |
| 24 | Albertini | Yes | Yes | Yes | No | Yes | Yes | No | No | No | No | 5 |
| 25 | Gokhale | Yes | Yes | Yes | No | Yes | No | No | Yes | No | Yes | 6 |
| 20 | Rosas | Yes | Yes | Yes | Yes | Yes | Yes | Yes | Yes | Yes | Yes | 10 |
| 21 | Hermine | Yes | Yes | Yes | No | Yes | Yes | Yes | Yes | Yes | Yes | 9 |
| 22 | Veiga | Yes | Yes | Yes | No | Yes | Yes | Yes | Yes | No | Yes | 8 |
| 23 | Soin | Yes | Yes | Yes | No | Yes | Yes | Yes | Yes | No | Yes | 8 |
| 24 | Albertini | Yes | Yes | Yes | No | Yes | Yes | No | No | No | No | 5 |
| 25 | Gokhale | Yes | Yes | Yes | No | Yes | No | No | Yes | No | Yes | 6 |
